# Higher Accuracy of Arthroscopy Compared to MRI in the Diagnosis of Chondral Lesions in Acute Ankle Fractures: A Prospective Study

**DOI:** 10.3390/diagnostics14161810

**Published:** 2024-08-20

**Authors:** Ali Darwich, Dominik Nörenberg, Julia Adam, Svetlana Hetjens, Mohamad Bdeir, Andreas Schilder, Steffen Thier, Sascha Gravius, Ahmed Jawhar

**Affiliations:** 1 Department of Orthopedic and Trauma Surgery, University Medical Centre Mannheim, Medical Faculty Mannheim, University of Heidelberg, Theodor-Kutzer-Ufer 1–3, 68167 Mannheim, Germany; juliaadam@t-online.de (J.A.); mohamad.bdeir@umm.de (M.B.); thier@sportchirurgie-heidelberg.de (S.T.); sascha.gravius@umm.de (S.G.); jawhar_ahmed@yahoo.de (A.J.); 2 Department of Radiology and Nuclear Medicine, University Medical Centre Mannheim, Medical Faculty Mannheim, University of Heidelberg, Theodor-Kutzer-Ufer 1–3, 68167 Mannheim, Germany; dominik.noerenberg@medma.uni-heidelberg.de; 3 Institute of Medical Statistics and Biomathematics, University Medical Centre Mannheim, Medical Faculty Mannheim, University of Heidelberg, Theodor-Kutzer-Ufer 1–3, 68167 Mannheim, Germany; svetlana.hetjens@medma.uni-heidelberg.de; 4 ATOS-Clinic Heidelberg, Bismarckstrasse 9-15, 69115 Heidelberg, Germany

**Keywords:** incidence, ICRS classification, size, chondral lesions, ankle fracture, MRI, arthroscopy

## Abstract

Even after successful surgery for acute ankle fractures, many patients continue having complaints. A possible explanation is the presence of concomitant chondral lesions. The aim of this study is to investigate the accuracy of MRI compared to that of arthroscopy in the assessment of chondral lesions in acute ankle fractures. In this prospective single-center study, patients presenting with acute ankle fractures over a period of three years were identified. A preoperative MRI was performed within a maximum of 10 days after trauma. During surgery, ankle arthroscopy was also performed. The International Cartilage Repair Society (ICRS) cartilage lesion classification was used to grade the detected chondral lesions. To localize the chondral lesions, the talar dome was divided into eight zones and the tibial/fibular articular surfaces into three zones. In total, 65 patients (28 females) with a mean age of 41.1 ± 15 years were included. In the MRI scans, 70 chondral lesions were detected (69.2% of patients) affecting mostly the tibial plafond (30%) and mostly graded as ICRS 3. The mean lesion area measured was 20.8 mm^2^. In the arthroscopy, 85 chondral lesions were detected (70.8% of patients) affecting mostly the medial surface of the talar dome (25.9%) and mostly graded ICRS 3. The mean lesion area measured was 43.4 mm^2^. The highest agreement between the two methods was observed in the size estimation of the chondral lesions. The present study shows the reduced accuracy of MRI when compared to arthroscopy in the assessment of traumatic chondral lesions in the setting of acute ankle fractures especially regarding lesion size. MRI remains an essential instrument in the evaluation of such lesions; however, surgeons should take this discrepancy into consideration, particularly the underestimation of chondral lesions’ size in the preoperative planning of surgical treatment and operative technique.

## 1. Introduction

Ankle fractures are considered as some of the most common injuries of the lower limb, with a yearly incidence of 0.1 to 0.2% [1,2]. Open reduction and internal fixation (ORIF) of these unstable fractures delivers good to excellent outcomes and is considered to be the gold standard in terms of restoring the joint’s stability through a stable fixation after re-establishing an anatomical alignment [3,4]. Unfortunately, even after successful operative treatment, many patients continue to have complaints such as pain, recurrent swelling, and reduced range of motion [5,6].

A possible explanation for these persistent complaints is thought to be the presence of concomitant chondral lesions (CLs) that emerge in the setting of the acute ankle fracture. Trauma is believed to be the leading cause of CLs [7]; however, these CLs are often misdiagnosed or diagnosed late, which may lead not only to persistent pain and swelling but also, in 14 to 50% of cases, to joint degeneration and eventually posttraumatic osteoarthritis [8,9,10]. In fact, the ankle, in comparison with other lower limb joints, is considered as the joint in which posttraumatic osteoarthritis most commonly occurs [10].

In the literature, the incidence of CLs in acute ankle fractures is reported with a wide range of discrepancy. Two recent meta-analyses by Martijn et al. [11] and Darwich et al. [12] reported incidences of 45.1% and 58%, respectively, based on 19 studies included in each meta-analysis. This discrepancy is mainly due to the various diagnostic methods used in the identification and evaluation of CLs. For instance, in the meta-analysis of Darwich et al. [12], the incidence of CLs increased to 65% based on the studies using arthroscopy as a diagnostic method and decreased to 19% based on the studies using magnetic resonance imaging (MRI). Another factor for this discrepancy is the heterogeneity of the studies regarding the included articular surfaces; some authors only examined CLs of the talar dome [13], while others included all articular surfaces of the ankle including the tibia and the fibula [14].

The advantages of an additional arthroscopy in the surgical treatment of ankle fracture have been reported in several studies. Smith et al. [15] compared outcomes after ankle fracture fixation with or without ankle arthroscopy and observed significant improvement in patient-reported outcomes for Weber B fibula fractures and ankle dislocations when additional arthroscopy was performed. Similarly, Baumbach et al. [16] observed better outcomes in patients with arthroscopically assisted internal fixation of ankle fractures in a propensity score-matched analysis and Liu et al. [17] came to the same conclusion with better outcomes in patients with arthroscopically assisted internal fixation of isolated fractures of the medial malleolus. The main advantage of the arthroscopy was reported to be the ability to detect concomitant injuries such as osteochondral lesions, partial-thickness cartilage injury, ligamentous injuries, or loose bodies in the joint and address them [15].

The value of MRI in the diagnosis of concomitant CLs in the setting of acute ankle fractures, especially the evaluation of the subchondral situation, has also been investigated. Boraiah et al. [13] retrospectively analyzed the MRI scans of 153 patients with ankle fractures and observed concomitant CLs of the talar dome in 17% of cases. Kortekangas et al. [18] observed concomitant CLs of all articular surfaces of the ankle joint in 54% of cases.

On the other hand, discrepancies in the assessment of these CLs were also repeatedly reported. Mintz et al. [19] reported an underestimation of the extent of CLs in MRI, and Lee et al. [20] and Bae et al. [21] reported an underestimation of the staging of talar CLs in MRI.

However, none of these studies performed an arthroscopy and an MRI scan on the same patient cohort in the sense of a direct comparison to assess the accuracy of each of the diagnostic tools in the detection of these important concomitant CLs. This information is of great value as the extent of these lesions is essential in the choice of treatment option and surgical decision making, hence the great importance of an accurate preoperative CL assessment [22].

The aim of this prospective study is to investigate the accuracy of MRI scans in the assessment of CLs in acute ankle fractures in comparison with intraoperative arthroscopic findings especially regarding the evaluation of the size and extent of chondral damage. We hypothesized that the preoperative MRI evaluation tends to underestimate the extent of CLs compared to the arthroscopic findings.

## 2. Materials and Methods

### 2.1. Study Patients

In this prospective single-center study, all patients presenting with an incongruent or unstable ankle fracture over a period of three years were identified. Excluded were patients with rheumatoid arthritis or osteoarthritis of the ankle joint as well as patients with open fractures, additional injuries in the same extremity, or polytrauma. Also excluded were pathological fractures due to an underlying malignancy and patients with previous ankle surgeries or an active infection. The exclusion criteria also involved patients not able to provide a written consent and patients with an intellectual disability or language disorder preventing them from fully understanding the trial features. All the remaining patients were included.

The current study included 65 ankle fractures in 65 patients (37 males (56.9%) and 28 females (43.1%)). The mean age of the included patients at the time of surgery was 41.1 ± 15 years (range 15–69 years) and the mean body mass index (BMI) was 26.9 ± 5.1 kg/m^2^ (range 19.1–45 kg/m^2^). In all, 66.2% of the patients (43/65 patients) were smokers. The mean time between trauma and MRI was 5 ± 3.8 days (range 0–10 days), and the time between MRI and surgery was 3.5 ± 2.4 days (range 0–8 days). In 33 cases (51%), the right side was involved, and in 32 cases (49%), it was the left side. Furthermore, 50 cases (77%) involved the lateral malleolus; 10 cases (15%) were trimalleolar fractures; 3 cases (5%) were considered as Maisonneuve fractures; and 2 cases (3%) were isolated fractures of the posterolateral tibial lip (Volkmann’s triangle).

The current study is a part of a larger-scale prospective clinical study. The data concerning the MRI evaluation were partially employed by our research group to address a different question and investigate a different hypothesis.

### 2.2. Radiological Assessment

In all the included patients, a preoperative MRI scan was performed within a maximum of 10 days after trauma. The scan was completed using the following protocol to assess cartilage involvement including prevalence, grade, and location (Table 1).

A 1.5 T MRI scanner (Magnetom Sola, Siemens Healthineers, Erlangen, Germany) within our institution was used to perform all the scans. With the use of a phased-array foot-and-ankle coil with 16 channels, the examinations were conducted with the patients in a supine position and the ankles in a neutral position.

**Figure 1:**
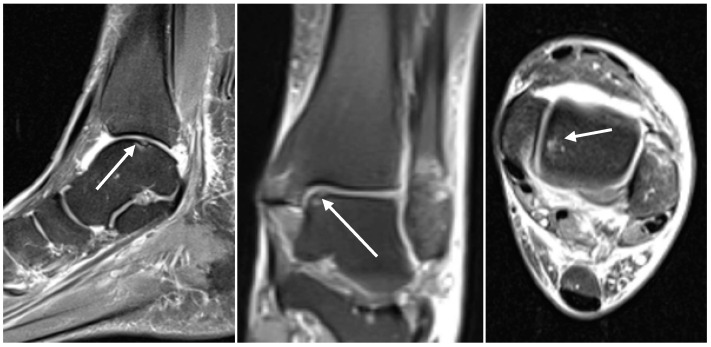
Sagittal (**left**), coronal (**middle**), and axial (**right**) proton-density-weighted turbo-spin-echo fat-saturation MRI sequences (PD tse fs) showing CLs (white arrows) of the talar dome in a 59-year-old female with an acute ankle fracture.

An experienced board-certified radiologist (D.N.) specialized in musculoskeletal MRI with 10 years of experience evaluated the acquired images. The reviewer was blinded to the clinical findings of the patients and used an image processing software (Osirix DICOM viewer Version v.3.9.4 64-bit (Pixmeo, Geneva, Switzerland)) to measure the CLs on the performed MRI scans. For measurements, the largest lesion diameter in the coronal and sagittal planes was utilized, and the depth was determined from the rim of the surrounding cartilage layer to the base of the lesion (Figure 1). To calculate the lesion area, the elliptical area formula described by Choi et al. [23] was used.

The images were also evaluated by an experienced board-certified trauma surgeon (A.D.) with 10 years of experience blinded to the results of the radiologist.

### 2.3. Arthroscopic Evaluation

In all the included patients, an operative treatment according to the Arbeitsgemeinschaft Für Osteosynthesefragen (AO) principles [24] was performed and involved ORIF. Before ORIF, an ankle arthroscopy was also performed in all the included patients. All the arthroscopies were performed by the senior author (A.J.).

The patients were positioned in the supine position. A bump was inserted under the hip. Distraction of the ankle was not required in any of the cases, since the instability of the fracture offered an easier joint entry. The arthroscopy was performed via standard anteromedial, anterolateral, and posterolateral portals [25] using a 2.7 mm, 30° scope (Karl Storz, Tuttlingen, Germany). Under direct visualization with the scope, the widest diameter of the lesion in 2 planes was identified and used for measurement. Accurate measurement was performed using a graduated probe with 1.0 mm graduations. Measurements were performed after complete debridement of the lesion and removal of possible unstable parts and cartilage flaps on the periphery of the remaining lesion.

### 2.4. Assessment of Chondral Lesions

The International Cartilage Repair Society (ICRS) cartilage lesion classification system was used to grade the detected lesions [26,27,28]:

- Grade 0: Normal.
- Grade 1: Superficial lesions. Soft indentation (A) and/or superficial fissures and cracks (B).
- Grade 2: Lesions extending down to <50% of cartilage depth.
- Grade 3: Cartilage defects extending down to >50% of cartilage depth (A) as well as down to the calcified layer (B) and down to but not through the subchondral bone (C). Blisters are included in this grade (D).
- Grade 4: Severely abnormal. Complete cartilage lesion with perforation of the subchondral plate.

**Figure 2:**
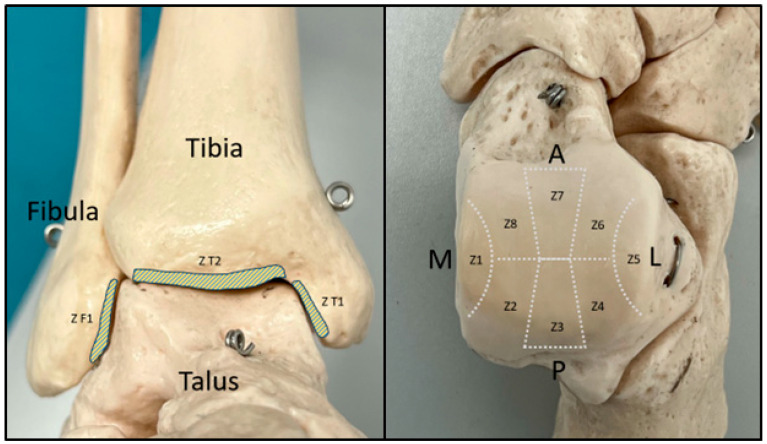
Zone distribution based on the schematic pattern proposed by Leontaritis et al. [8] in frontal view of the ankle (**left**) and axial view of the talus (**right**) (Z, zone; M, medial; L, lateral; A, anterior; P, posterior).

The schematic pattern proposed by Leontaritis et al. [8] was used to document the localization of the detected lesions. In this pattern, the talar dome is divided into 8 zones (Z1 to Z8), and the articular surfaces of the tibia and fibula are also divided into 3 zones (ZT1, ZT2, and ZF1) (Figure 2).

### 2.5. Statistical Analysis

All the analyses were performed using SAS (Version 9.4 SAS Institute Inc., Cary, NC, USA). The qualitative factors are presented in the form of absolute and relative frequencies. The mean values and standard deviations (±SDs) or medians with interquartile range (IQR) were calculated to present quantitative variables. To measure agreement and compare results, the kappa coefficient κ was calculated. The kappa coefficient κ values were interpreted according to Landis et al. [29]. Statistical significance was assumed for *p* values less than 0.05.

### 2.6. Ethics Approval

This study was performed in line with the principles of the Declaration of Helsinki. Approval for this prospective analysis was granted by the ethics committee of clinical research at our institution (Ethikkommission II, University Medical Centre Mannheim, Medical Faculty Mannheim, Heidelberg University, Theodor-Kutzer-Ufer 1–3, 68167, Mannheim, approval No. 2016-509N-MA).

## 3. Results

### 3.1. Assessment of the Preoperative MRI Scans

In the preoperative MRI scans, 45 of the 65 included patients (69.2%) showed signs of CLs. Overall, 7 patients had 3 CLs each; 11 patients had 2 CLs each; and 27 patients had 1 CL each, making a total of 70 CLs detected. There were no significant CLs detected in the MRI scans of the remaining patients, even though the included fractures are formally intraarticular in nature (Table 2).

In 22 of the 45 patients with CLs (48.9%), the lesions involved only the talar dome, and in 10/45 patients (22.2%), the CLs involved only the tibial articular surface. In 13/45 patients (28.9%), the CLs were shown on both articular surfaces. The fibular articular surface did not show any significant CLs.

**Figure 3:**
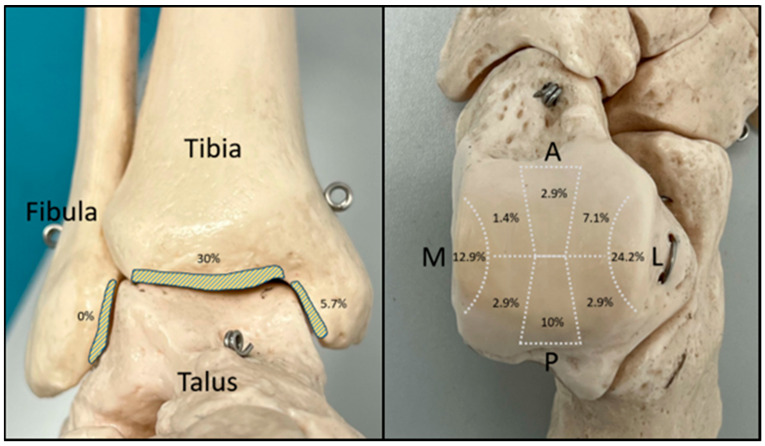
Zone allocation of the detected CLs in the preoperative MRI (M, medial; L, lateral; A, anterior; P, posterior).

The most commonly affected zone was the articular surface of the tibial plafond, with 30% of the total CLs detected. On the talar dome, the lateral surface was mostly affected with 24.2% of the detected CLs followed by the medial articular surface (12.9%). A detailed description of the zone allocation of the detected CLs can be found in Figure 3.

Concerning the size of the detected CLs, a mean lesion area of 20.8 ± 12.8 mm2 was measured. Regarding the ICRS grading of the detected CLs, most of the identified CLs (38.6%) (27 of a total of 70 CLs) were cartilage defects extending down to > 50% of cartilage depth and were graded as ICRS 3a. Furthermore, 21 of the 70 identified CLs (30%) were graded ICRS 2; 18 of 70 (25.8%) were graded ICRS 4; and 4 of 70 (5.6%) were graded ICRS 1b. In 36 of the 70 CLs identified, subchondral edema was also detected (Table 3).

### 3.2. Evaluation of the Intraoperative Arthroscopic Findings

In total, 46 of the 65 included patients (70.8%) showed signs of CLs in the performed arthroscopy. Of these, 30 patients had 1 CL each; 9 patients had 2 CLs each; 2 patients had 3 CLs each; 2 patients had 5 CLs each; 1 patient had 6 CLs; 1 patient had 7 CLs; and 1 patient had 8 CLs, making a total of 85 CLs detected (Table 4). Intraoperatively, there were no significant CLs detected in the remaining patients. In 37 of the 46 patients with CLs (80.4%), the lesions involved only the talar dome, and in one patient (2.2%), the CLs involved only the tibial articular surface. In 6/46 patients (13%), the CLs were shown on both talar and tibial articular surfaces, and in 2/46 patients (4.3%), the CLs were shown on both talar and fibular articular surfaces. 

**Figure 4:**
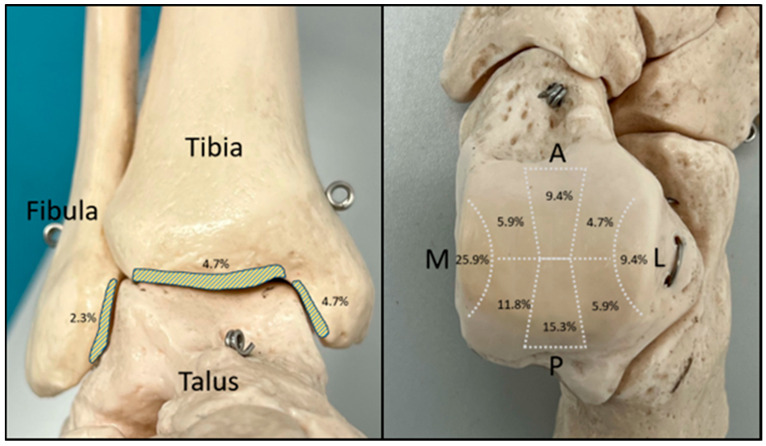
Zone allocation of the detected CLs in the arthroscopy (M, medial; L, lateral; A, anterior; P, posterior).

Overall, 4.7% of the identified CLs were found on the articular surface of the tibial plafond and 4.7% on its medial surface. The fibular articular surface was the least involved, with only 2.3% of the detected CLs. On the talar dome, the medial surface was mostly affected with 25.9% of the identified CLs followed by the posterior articular surface (15.3%) (Figure 4).

Concerning the size of the detected CLs, a mean lesion area of 43.4 ± 29.7 mm^2^ was measured. Regarding the ICRS grading, 18 of the 85 (21.2%) total identified CLs were graded as ICRS 4, followed by 28 of 85 (32.9%) graded ICRS 3, 24 of 85 (28.2%) graded ICRS 2, and 15 of 85 (17.6%) as ICRS 1 (Table 5).

### 3.3. Validation

The interobserver agreement between the two reviewers of the MRI scans was variable according to the zone being evaluated and ranged from moderate to slight with kappa values ranging from κ = 0.4227 to κ = 0.1384. A detailed analysis was presented in the study of Darwich et al. [30]. The presented data in the current study are the results of the evaluation of the radiologist.

The agreement analysis between the preoperative MRI evaluation and the intraoperative arthroscopic findings showed slight-to-fair agreement in CL identification in talar zones 1, 2, 5, and 7 (κ = 0.3825, *p* = 0.0005; κ = 0.1237, *p* = 0.0114; κ = 0.1905, *p* = 0.0290; and κ = 0.1605, *p* = 0.0339, respectively). 

Regarding the ICRS classification of the detected CLs, the analysis showed no agreement between the two modalities. 

Concerning the size evaluation of the detected CLs, a significant agreement was shown in the size estimation of the CLs in talar zones 1, 2, 3, and 7 (*p* < 0.0001, *p* = 0.0313, *p* = 0.0108, and *p* = 0.0273, respectively) as well as in the tibial articular surface T2 (*p* = 0.0004) (Table 6).

## 4. Discussion

The choice of the most appropriate surgical treatment of cartilage lesions in the setting of ankle fractures relies largely on the exact estimation of the size, localization, and extent of these lesions, which eventually allows the improvement of the long-term outcome [13]. Due to its superior soft tissue visualization and its high sensitivity/specificity in the diagnosis of cartilage lesions as well as its ability to assess not only osteochondral damage but also deeper bone affection and surrounding soft tissue structures, many authors regard MRI as an essential tool in the preoperative planning of such injuries [31,32].

On the other hand, several studies have shown discrepancies in the findings of the preoperative MRI scans when compared to the intraoperative arthroscopic findings [19,20,21,33,34,35]. In the current study, the authors hypothesized that the preoperative MRI scan in the setting of acute ankle fractures underestimates the incidence and extent of concomitant CLs.

**Figure 5:**
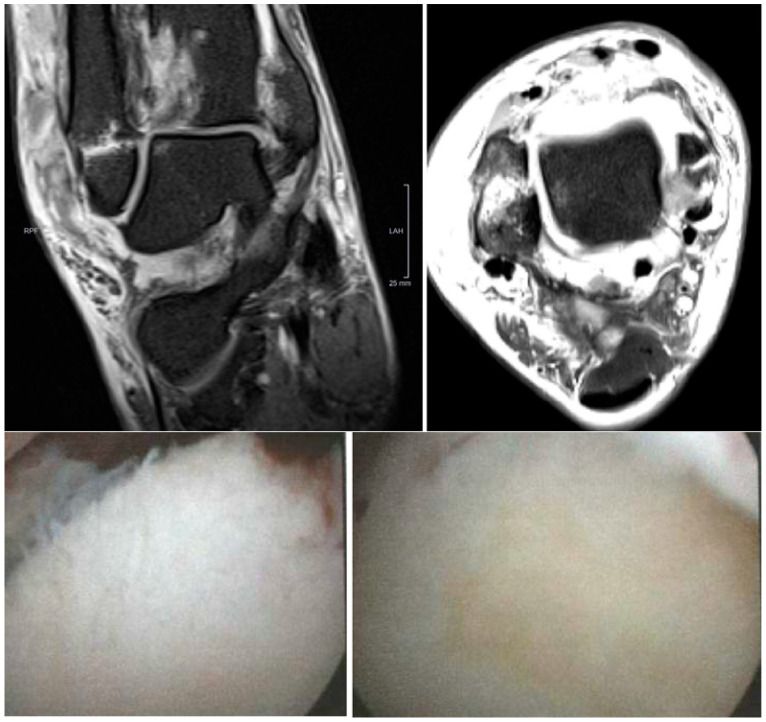
Coronal (**above left**) and axial (**above right**) proton-density-weighted turbo-spin-echo fat-saturation MRI sequences (PD tse fs) showing a 4 × 4 mm ICRS 1b CL in zone 5 of the talar dome, with subchondral edema in a 31-year-old male with an acute ankle fracture. Below are the intraoperative figures of the corresponding lesions in arthroscopy in zone 5 of the talar dome measuring 8 × 10 mm and grading ICRS 2.

**Figure 6:**
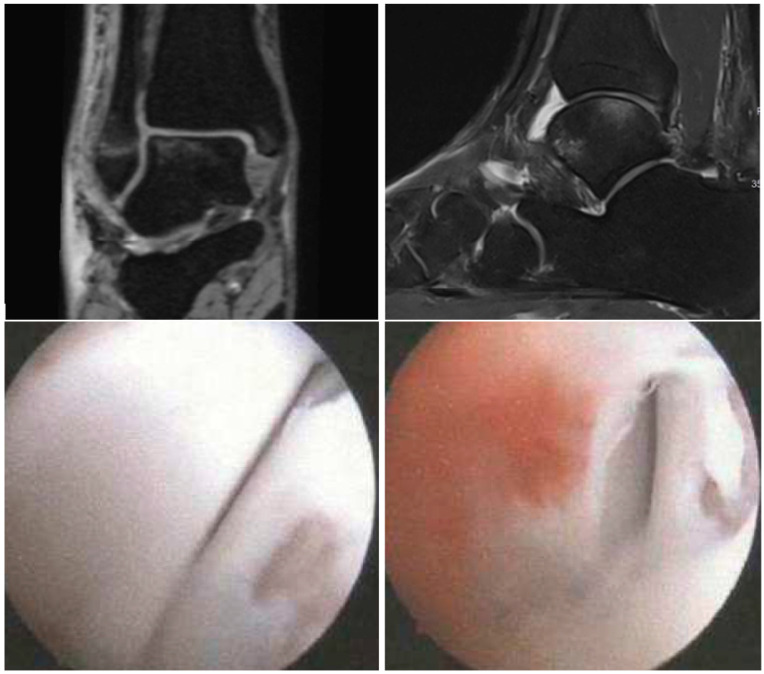
Coronal (**above left**) T2 3D-double-echo with water excitation (T2 DE3D WE) and sagittal (**above right**) proton-density-weighted turbo-spin-echo fat-saturation (PD tse fs) MRI sequences showing a 3 × 3 mm ICRS 3a CL in zone 5 of the talar dome with subchondral edema in a 20-year-old female with an acute ankle fracture. Below are the intraoperative figures of the corresponding lesions in arthroscopy in zone 5 of the talar dome measuring 6 × 6 mm and grading ICRS 3b.

In the present study, 82.4% of the CLs identified arthroscopically were detected in the preoperative MRI scan. The grading of the CLs tended to be underestimated in the MRI: 15 lesions were staged ICRS 1 in the arthroscopy versus only 4 in the MRI; 24 were graded as ICRS 2 in the arthroscopy versus 21 in the MRI; and 46 lesions were staged ICRS 3-4 in the arthroscopy versus 45 in the MRI (Figure 5 and Figure 6). However, no statistically significant agreement between the grading of the CLs in each zone between MRI and arthroscopy could be observed.

Mintz et al. [19] retrospectively reviewed the data of 54 patients undergoing ankle MRI and arthroscopy. The performed MRI was able to identify 100% of the lesions detected intraoperatively but graded only 83% of them correctly and underestimated the extent of the remaining 17% of the lesions. In their study, the included patients presented mainly with osteochondritis dissecans and chondral ankle injury. The evaluation of lesions involved only the talar dome and not the entire ankle joint, and the mean interval between the preoperative MRI scan and the surgery was 84 days. Lee et al. [20] prospectively analyzed the data of 50 patients undergoing ankle MRI and arthroscopy and observed an MRI accuracy of 81% in staging osteochondral lesions of the talus. The stage of the remaining lesions was underestimated in the preoperative MRI scan. In their study as well, the evaluation of lesions involved only the talar dome. Bae et al. [21] retrospectively reviewed the data of 40 patients with osteochondral lesion of the talus and found an agreement in the staging of the lesions between both MRI and arthroscopy for 65.9% of the lesions. The preoperative MRI underestimated the lesion staging in 20.5% of the lesions and overestimated it in 13.6%. In their study also, the evaluation of lesions involved only the talar dome, and the mean interval between the preoperative MRI scan and the arthroscopy was 2.7 months. Dipaola et al. [33] analyzed the data of 12 patients undergoing preoperative MRI and arthroscopy. Among these cases, six involved the ankle joint. In 5/6 (83%) cases, the MRI scan correctly staged the lesion extent, and in 1/6 (17%), the lesion’s stage was overestimated. In their study, the MRI was performed using a 0.35 Tesla magnet. The evaluation involved only six patients and included only the talar dome. 

The lack of agreement between MRI and arthroscopy was also observed in several other studies, in which a wide range of agreement rates from as low as 65.9% to as high as 92% were reported regarding CL grading between these two modalities [19,20,21,33,36,37,38]. On the other hand, the clinical relevance of the exact grading of these lesions remains debatable, as several authors showed the absence of agreement between the grading of CLs in MRI and the clinical outcome of the patients [25,39]. In a case series of 50 patients with osteochondral lesions of the talus, Ferkel et al. [25] observed no agreement between MRI grading and clinical outcome using Alexander, modified Weber, and American Orthopaedic Foot and Ankle Society (AOFAS) Ankle/Hindfoot scores. Similarly, Choi et al. [39] found no agreement in their prospective study including 120 ankles between the MRI, CT, or plain radiograph grading and the clinical outcome measured with the AOFAS) Ankle/Hindfoot score. In addition, high-grade CLs (ICRS 3 and 4) were consistently detected in both modalities in the present study (45 CLs in MRI and 46 CLs under arthroscopy). These unstable cartilage injuries are of great concern for the surgeon since they may often require debridement and additional surgical therapy [40].

Concerning the size of the detected CLs, in the current study, a mean lesion area of 43.4 ± 29.7 mm^2^ was measured arthroscopically, and it was 20.8 ± 12.8 mm^2^ in the preoperative MRI scans. An underestimation of the lesion size in MRI was observed in 63.3% of CLs. 

Yasui et al. [35] analyzed the data of 45 osteochondral lesions of the ankle in 39 patients undergoing MRI and arthroscopy and observed an underestimation of the size of the lesions in 24.4% of ankles in the MRI. This discrepancy in comparison to the results of the current study may be due to the fact that the measurement of the lesions intraoperatively was carried out prior to the debridement of any unstable cartilage and not after debridement. Fragmented and unstable fragments surrounding the CLs are typically visualized in the arthroscopy and not identified in MRI, which may lead to such discrepancies in the lesion assessment [26,36].

Campbell et al. [36] compared the preoperative MRI findings with the post-debridement arthroscopy findings in 77 patients with knee articular cartilage defects and observed an underestimation of the size of the defect area in 70% of cases on average. Similarly, Gomoll et al. [37] retrospectively investigated the data of 38 patients with knee articular cartilage defects and observed an underestimation of the size of the defect area in 85% of cases in the preoperative MRI, when compared to intraoperative findings.

Apart from the effect of intraoperative debridement on the discrepancy in lesion size evaluation, the geometrical configuration of the ankle joint and its articular surface may also play a role in the misestimation of the lesion size, especially when it comes to lesions on the talar dome, whose convexity negatively affects the visualization ability of axial slices [35]. 

### Study Limitations 

The limited number of included patients may have reduced the statistical significance of some of the agreements measured. Another limitation is the use of ankle arthroscopy by itself as a diagnostic instrument, where a certain reduction of the visualization due to restricted ankle mobility and the need of multiple portals has been reported [41].

In addition, a supplementary interobserver evaluation of the arthroscopy measurements would have been beneficial.

Another limitation is the use of the ICRS classification in the evaluation of chondral lesions in the MRI scans. This classification score was originally developed based on arthroscopic evaluation; however, its use has been extended to MRI-based cartilage evaluation as well [42,43,44,45].

A further limitation may be the 1.5 T MRI scanner used in the current study. The literature data show a higher diagnostic performance for 3 T MRI scanners when compared to 1.5 T scanners especially in the evaluation of joint cartilage. Chopra et al. [46] showed a higher diagnostic accuracy for 3 T MRI scanners in comparison with 1.5 T scanners in the detection of cartilage defects in the hip joint of 68 patients with suspected femoroacetabular impingement. Similarly, Cheng et al. [47] showed, in a meta-analysis including 16 studies, the greater diagnostic accuracy of 3 T MRI scanners when compared to 1.5 T scanners in the detection of articular cartilage lesions of the knee. Comparable data involving the ankle joint are not available; however, the results of the above-mentioned studies suggest that the discrepancies between MRI and arthroscopy observed in the current study may have been partly reduced with the usage of a higher-field MRI scanner.

Nevertheless, this is the first prospective study to investigate CLs in the setting of acute ankle fractures using both MRI and arthroscopy. In addition, all the MRI scans were performed within 10 days after the trauma, which excludes the possibility of relevant cartilage damage progression between the MRI and surgery and minimizes the degree of confounding.

## 5. Conclusions

The present study shows the reduced accuracy of MRI when compared to arthroscopy in the assessment of traumatic CLs in the setting of acute ankle fractures, especially regarding lesion size. MRI remains an essential instrument in the evaluation of such lesions; however, surgeons should take this discrepancy into consideration, particularly the underestimation of CL size in the preoperative planning of surgical treatment and operative technique. Based on the results of this study regarding the superiority of arthroscopy in the proper assessment of chondral lesion in the setting of acute ankle fractures, the recommendation to complement the surgical fixation with arthroscopy and allocate more resources from healthcare systems to support this approach may be warranted. Another way to try reducing this discrepancy between MRI and arthroscopy may be the use of higher-field MRI scanners of 3T, 7T, or even more advanced technologies to improve the MRI evaluation of these lesions.

## Figures and Tables

**Table 1:** MRI protocol used.

Sequence	Fat-Saturated Proton-Density-Weighted Turbo Spin Echo (PD TSE)	T1-Weighted Turbo Spin Echo (T1 TSE)	T2-Weighted Turbo Spin Echo (T2 TSE)
Slice thickness (in mm)	2 (coronal, sagittal) 3 (transversal)	2 (coronal)	3 (transversal)
TR (in ms)	3470–4000	556	5000
TE (in ms)	40–47	12	73
Field of view (FOV) (in cm)	14	14	14
Matrix	512 × 384	512 × 384	512 × 384

**Table 2:** Detected CLs in the preoperative MRI.

Number of CLs Detected	1	2	3	Total
Number of patients	27	11	7	45 patients
Total	70 CLs			

**Table 3:** Zone distribution of the detected CLs in MRI and their size and ICRS classification as well as the presence of subchondral edema.

Zone	Mean Size (mm^2^)	ICRS Classification					Subchondral Edema	Total
		1a	1b	2	3a	4		
1	23.78	-	1	4	2	2	2	9
2	40	-	-	-	1	1	1	2
3	14.86	-	-	3	4	-	3	7
4	17.5	-	-	1	1	-	0	2
5	13.71	-	2	6	9	-	7	17
6	11.4	-	1	2	2	-	3	5
7	9	-	-	1	1	-	0	2
8	35	-	-	-	-	1	0	1
F1	-	-	-	-	-	-	0	0
T1	26.75	-	-	1	2	1	1	4
T2	36.81	-	-	3	5	13	19	21
Total	20.8	0	4	21	27	18	36	70

**Table 4:** Detected CLs in the arthroscopy.

Number of CLs Detected	1	2	3	5	6	7	8	Total
Number of patients	30	9	2	2	1	1	1	46 patients
Total	85 CLs							

**Table 5:** Zone distribution of the detected CLs in arthroscopy and their size and ICRS classification.

Zone	Mean Size (mm^2^)	ICRS Classification							Total
		1a	1b	2	3a	3b	3c	4	
1	42.09	-	-	13	4	-	2	3	22
2	76.7	2	-	1	3	-	2	2	10
3	47.15	-	2	6	3	-	-	2	13
4	19.8	-	1	-	2	-	-	2	5
5	13.38	1	2	1	3	1	-	-	8
6	116.25	-	-	-	2	-	-	2	4
7	22.63	3	1	2	1	-	-	1	8
8	40.8	-	-	-	3	1	-	1	5
F1	29.5	1	-	-	-	-	-	1	2
T1	25.75	1	-	-	-	-	-	3	4
T2	43.75	-	1	1	-	1	-	1	4
Total	43.4	8	7	24	21	3	4	18	85

**Table 6:** Agreement between the preoperative MRI evaluation and the arthroscopic findings regarding chondral lesions’ (CLs’) identification, their size, and their ICRS classification in each zone.

Zone	CL Identification		ICRS Classification		Size Evaluation
	Kappa	*p* Value	Kappa	*p* Value	*p* Value
1	*0.3825*	*0.0005*	0.3196	0.3955	*<0.0001*
2	*0.1237*	*0.0114*	0.1403	0.9576	*0.0313*
3	0.1919	0.1088	0.1768	0.8713	*0.0108*
4	0.2544	0.1797	0.1207	0.9473	0.2188
5	*0.1905*	*0.0290*	0.1094	0.9159	0.0991
6	−0.0699	0.7389	−0.0479	0.9856	0.4063
7	*0.1605*	*0.0339*	0.0741	0.8775	*0.0273*
8	−0.0251	0.1025	−0.0149	0.6767	0.2188
T1	0.2031	1.000	0.0933	0.9311	0.9844
T2	*0.0679*	*0.0002*	0.0650	0.2137	*0.0004*
F1	0	0.1573	0	0.1573	1

## Data Availability

Data supporting the findings of this study are available from the corresponding author [A.D.] on request.
